# *Drosophila* sensory cilia lacking MKS proteins exhibit striking defects in development but only subtle defects in adults

**DOI:** 10.1242/jcs.194621

**Published:** 2016-10-15

**Authors:** Metta B. Pratt, Joshua S. Titlow, Ilan Davis, Amy R. Barker, Helen R. Dawe, Jordan W. Raff, Helio Roque

**Affiliations:** 1 The Sir William Dunn School of Pathology, University of Oxford, South Parks Road, Oxford OX1 3RE, UK; 2 Department of Biochemistry, University of Oxford, South Parks Road, Oxford OX1 3QU, UK; 3 Centre for Microvascular Research, William Harvey Research Institute, Queen Mary University of London, London EC1M 6BQ, UK; 4 Biosciences, College of Life and Environmental Sciences, University of Exeter, Exeter EX4 4QD, UK

**Keywords:** Ciliogenesis, MKS module, Sensory cilia, Transition zone

## Abstract

Cilia are conserved organelles that have important motility, sensory and signalling roles. The transition zone (TZ) at the base of the cilium is crucial for cilia function, and defects in several TZ proteins are associated with human congenital ciliopathies such as nephronophthisis (NPHP) and Meckel–Gruber syndrome (MKS). In several species, MKS and NPHP proteins form separate complexes that cooperate with Cep290 to assemble the TZ, but flies seem to lack core components of the NPHP module. We show that MKS proteins in flies are spatially separated from Cep290 at the TZ, and that flies mutant for individual MKS genes fail to recruit other MKS proteins to the TZ, whereas Cep290 seems to be recruited normally. Although there are abnormalities in microtubule and membrane organisation in developing MKS mutant cilia, these defects are less apparent in adults, where sensory cilia and sperm flagella seem to function quite normally. Thus, localising MKS proteins to the cilium or flagellum is not essential for viability or fertility in flies.

## Abstract (precis)

**Highlighted Article:** An analysis of Meckel–Gruber syndrome (MKS) fly mutants reveals that cilium structure is perturbed during development, but that cilia are largely structurally and functionally normal by adulthood.

## INTRODUCTION

Cilia and flagella are microtubule (MT)-based extensions of the plasma membrane present in evolution since the last eukaryotic common ancestor (Ishikawa and Marshall, 2011; Nigg and Raff, 2009; Sung and Leroux, 2013). Cilia have diverse roles in cellular sensation, signalling and motility (Basten and Giles, 2013; Berbari et al., 2009; Nigg and Raff, 2009) and are formed when the centriole pair migrates to the plasma membrane (PM). The older, mother, centriole forms a basal body (BB) that docks at the PM and MTs extend from the distal end of the BB to form a membrane-bounded axoneme. Concomitantly, many proteins are recruited to the transition zone (TZ), a complex structure assembled where the BB meets the PM (Garcia-Gonzalo and Reiter, 2012; Ishikawa and Marshall, 2011). The TZ is thought to be essential for cilium function and helps assemble a membrane and cytoplasmic barrier that allows the cilium to form a distinct cellular compartment (Czarnecki and Shah, 2012; Hsiao et al., 2012; Hu and Nelson, 2011; Nachury et al., 2010; Reiter et al., 2012).

Interestingly, many of the genes linked to cilia dysfunction in humans, such as those associated with Meckel–Gruber syndrome (MKS), Joubert syndrome (JBT) and nephronophthisis (NPHP), encode proteins that localise to the TZ (Czarnecki and Shah, 2012; Davis and Katsanis, 2012; Hu and Nelson, 2011; Reiter et al., 2012). Several studies have indicated that the TZ proteins function in broadly three distinct complexes or ‘modules’; an MKS module (comprising proteins such as MKS1, Tectonic, B9D1 and B9D2), an NPHP module (comprising proteins such as NPHP1 and NPHP4) and a Cep290 module (comprising proteins such as CEP290) although there is variability and some overlap of module components depending on cell type and species (Garcia-Gonzalo et al., 2011; Chih et al., 2011; Sang et al., 2012; Schouteden et al., 2015; Yee et al., 2015; Williams et al., 2008, 2011; Winkelbauer et al., 2005). Proteins of one module are generally required for the recruitment of other members of the same module, whereas the recruitment of each module can occur at least partially independently of the other modules (Basiri et al., 2014; Bialas et al., 2009; Schouteden et al., 2015; Williams et al., 2008, 2011).

Studies, primarily in worms, indicate that there is a modular hierarchy of TZ assembly with NPHP8 (also known as RGRIP1L and MKS5) (Jensen et al., 2015; Li et al., 2016) initially recruiting Cep290 to form the central cylinder of the TZ (Schouteden et al., 2015) and the MKS and NPHP modules then cooperating to form the Y-links (Williams et al., 2008, 2011). Only when co-mutating or co-depleting components of both MKS and NPHP modules at the same time can a strong effect be observed (Dawe et al., 2007; Tammachote et al., 2009; Weatherbee et al., 2009; Williams et al., 2008, 2011; Yee et al., 2015). Interestingly, the same complex might perform slightly different functions in different organisms, or even different tissues, as observed in a mouse model of MKS1-associated MKS (Weatherbee et al., 2009).

The fruit fly *Drosophila melanogaster* has proved a powerful experimental system, yet comparatively little research has focused on the TZ in flies. The fly offers several advantages to study TZ function. Perhaps most importantly, flies do not use cilia for *hedgehog* or *wingless* signalling, so flies lacking cilia develop largely normally, without the gross morphological perturbations associated with defects in these signalling pathways in vertebrate systems (Basto et al., 2006). Indeed, most adult fly cells do not contain cilia or flagella, which are restricted to certain sensory neurons and sperm lineages (Kernan et al., 1994). Adult flies lacking centrioles and cilia are severely uncoordinated owing to the lack of cilia in their mechanosensory neurons and die shortly after eclosure (Basto et al., 2006). The analysis of TZ assembly and function is also potentially simplified in *Drosophila* as flies seem to lack a core NPHP module. Although two putative NPHP genes, *niki* (CG10951) and *Atxn10* (CG4975) were recently identified in flies (Basiri et al., 2014), no orthologues for the key NPHP module proteins, NPHP1, NPHP4 and NPHP8, have been found. In contrast, *Drosophila* orthologues of Cep290 and several members of the MKS module (including all but one of the ‘core’ MKS module proteins: TMEM67, CC2D2A, B9D1, B9D2, Tectonic and MKS1 – but not AHI1), have recently been identified (Barker et al., 2014; Basiri et al., 2014), suggesting that flies might rely only on the CEP290 and MKS modules for TZ assembly.

Moreover, it has recently been shown that Cep290 and Chibby (a conserved TZ protein involved in Wnt signalling in vertebrates, but not in flies) are required for cilia function in flies (Basiri et al., 2014; Enjolras et al., 2012). *Cep290* mutants are uncoordinated and although several MKS module proteins (MKS1, B9D1 and B9D2) were still recruited to the growing ciliary cap structure in elongating mutant spermatids, their localisation was abnormally diffuse (Basiri et al., 2014). *Cby* mutants exhibit reduced mechanosensation and male fertility, and have structural defects in their sensory neuron cilia and in the short primary cilia found in maturing spermatocytes as well as in the axonemes of mature sperm (Enjolras et al., 2012). The function of the conserved MKS module of proteins, however, has not been directly addressed in flies.

Here, we analyse the distribution of several MKS module proteins in flies and generate mutations in *Mks1* and *B9d1*. We show that the MKS-proteins are recruited to an area of the TZ that is spatially distinct from Cep290, and that mutations in *Mks1* and *B9d1* disrupt the TZ localisation of the other MKS proteins, supporting the idea that these proteins form a functional complex. Despite the lack of detectable MKS proteins at the TZ, *Mks1* and *B9d1* mutants are viable and fertile and, although MKS1 mutants exhibit structural defects at sensory cilia during development, these defects are largely absent in adults. Thus, even though flies lack an obvious NPHP module, they can still form functional cilia and flagella without an MKS module, given enough developmental time.

## RESULTS

### TZ proteins in *Drosophila melanogaster*

We previously performed a bioinformatics analysis of putative TZ proteins across species and identified a core conserved group of proteins that are present in >50% of ciliated organisms: AHI1, B9D1, B9D2, CC2D2A, Tectonic, TMEM67 and probably MKS1 (Barker et al., 2014). *Drosophila melanogaster* has identifiable homologues of all of these proteins except AHI1, and also has homologues of the TZ proteins Cep290, TMEM216, TMEM231 and TMEM237; no components of the core NPHP TZ module were identified (Barker et al., 2014). These findings are in broad agreement with another bioinformatics analysis of TZ proteins in *Drosophila*, although this study identified two putative non-core NPHP genes, *niki* (CG10951) and *Atxn10* (CG4975) (Basiri et al., 2014). In addition, Chibby (Cby) and Dilatory (Dila) have also recently been identified as components of the *Drosophila* TZ (Enjolras et al., 2012; Ma and Jarman, 2011).

### *Drosophila* TZ proteins occupy distinct regions in spermatocyte cilia

**Fig. 1.**
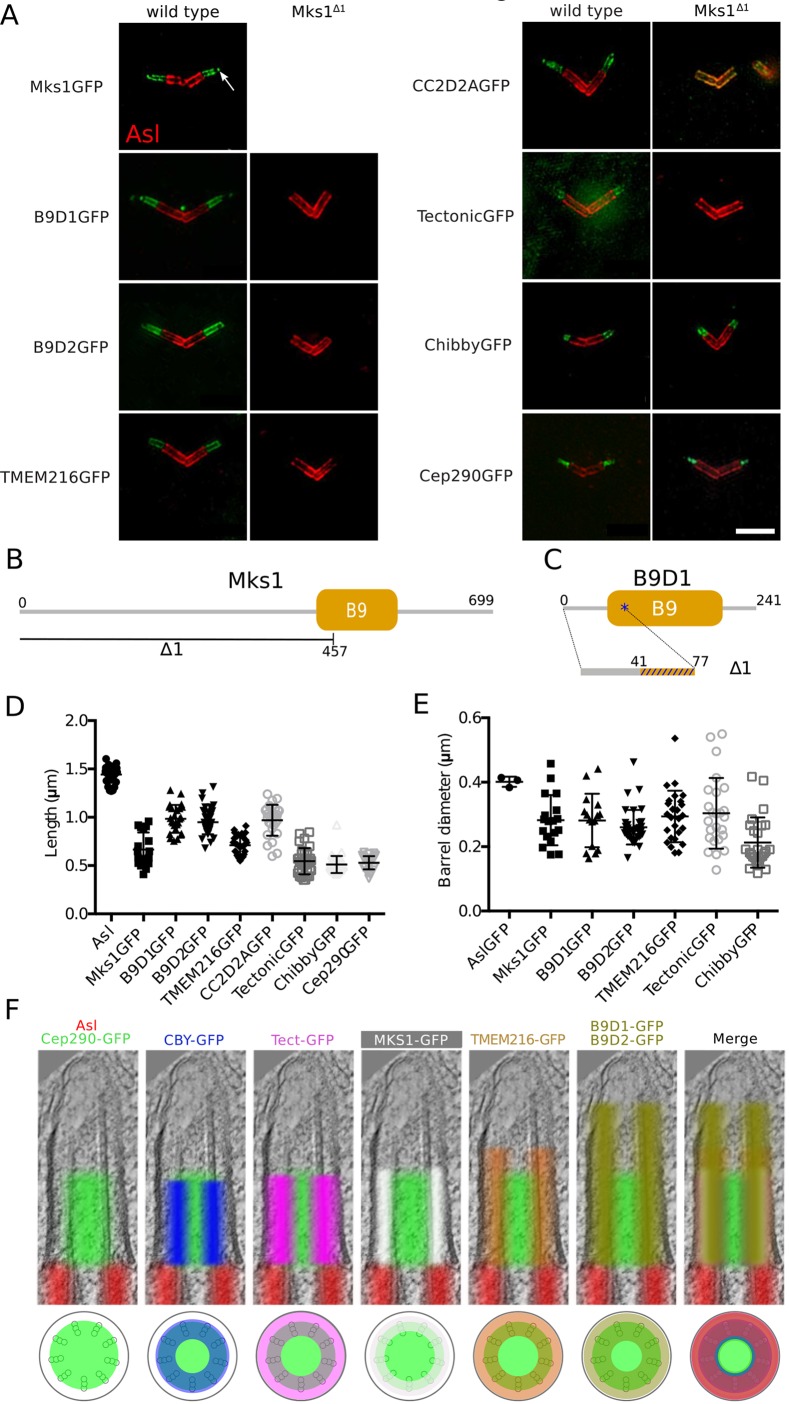
**MKS proteins occupy distinct regions within the TZ and depend on MKS1 for their TZ localisation.** (A) 3D-SIM micrographs of *Drosophila* spermatocyte cilia showing the localisation of various GFP-tagged TZ proteins (green, as indicated) in relation to the BB protein Asterless (Asl, red) in WT (left panels) and *Mks1*^Δ*1*^ mutants (right panels). (B,C) Schematic representations of (B) the *Mks1*^Δ*1*^ deletion and (C) the *B9d1*^Δ*1*^ mutation that creates a frameshift at amino acid 41 and a premature stop codon at amino acid 77 (blue asterisk). (D) Graph quantifies the longitudinal length of Asl and the TZ proteins in 3D-SIM micrographs. Isolated GFP dots, sometimes present at the distal tips of the TZ (arrows in A), were ignored for these measurements. (E) Graph quantifies the diameter of the ‘barrel’ formed by Asl and TZ proteins in 3D-SIM micrographs (see Materials and Methods). (F) Using the mean lengths and diameters obtained in D,E we created composite images (top panels) superimposing the localisation of Asl (red) and the TZ proteins (various colours, as indicated) on a typical *Drosophila* spermatocyte EM micrograph. A schematic of the transverse profile of the BB–axoneme complex (lower panel) shows the various protein localisations in relation to the ninefold symmetry MT blades (grey triplets) and the axoneme membrane (black circle). Error bars represent the s.e.m. Scale bar: 2 µm.

To better understand how TZ proteins are organised in *Drosophila* cilia we generated fly lines individually expressing GFP fusions to the core MKS module proteins MKS1, B9D1, B9D2, TMEM216, CC2D2A and Tectonic (see Materials and Methods). We also obtained lines expressing the TZ proteins Cep290–GFP (Basiri et al., 2014) and Chibby (Cby)–GFP (Enjolras et al., 2012). We used dual-colour 3D super-resolution structured illumination microscopy (3D-SIM) to image the distribution of each GFP-fusion at the BB–axoneme complex *in vivo* in fixed mature spermatocytes labelled with anti-Asterless (Asl) antibodies to mark the outer wall of the BB (Franz et al., 2013; Varmark et al., 2007) (Fig. 1A). These spermatocytes have long BBs that extend a short axoneme (A.D. Tates, PhD thesis, Rijksuniversiteit Leiden, The Netherlands, 1971) (Fig. S1), and several genes encoding TZ proteins are highly expressed in testes. Moreover, mutations in Cep290, Cby and Dila all lead to defects in spermatogenesis (Basiri et al., 2014; Enjolras et al., 2012; Ma and Jarman, 2011).

In wild-type (WT) spermatocytes all of the TZ proteins were located distally to the BB (stained by Asl, red in Fig. 1A) and extended into the axoneme to varying extents (Fig. 1A). We measured the average length and width of these axoneme extensions (Fig. 1D,E; note that for CC2D2A–GFP we could not accurately measure the width as its distribution was too irregular). We then created compound images (Fig. 1F) by overlaying the average distribution of each protein on an EM-tomogram of a typical WT BB–axoneme complex, aligning the markers so that the Asl staining terminated at the distal end of the BB (see Materials and Methods). These studies revealed that Cep290 occupied a distinct inner region of the TZ (shown in green in all the compound images) that seemed to overlap with the axonemal MTs. The distribution of Cby (blue) overlapped the outer portion of the Cep290 region, whereas Tectonic (purple) and MKS1 (white) occupied a region between the axonemal MTs and the ciliary membrane that was largely outside of the Cep290 region. TMEM216 (orange), B9D1 and B9D2 (both olive green) all also occupied a similar region between the axoneme and the membrane, but these regions extended distally beyond the other TZ proteins, with B9D1 and B9D2 extending the furthest. These studies demonstrate that individual TZ proteins occupy distinct regions within the TZ, in agreement with recent findings in cultured RPE-1 cells (Yang et al., 2015) as well as predictions based on the domain structure of these proteins (Garcia-Gonzalo et al., 2011). The localisation patterns of individual TZ proteins, including Cep290, MKS1, B9D1, B9D2, Cby observed here are in broad agreement with earlier studies (Basiri et al., 2014; Lee et al., 2014).

### MKS1 and B9D1 are required to localise the MKS module to the TZ

To study the function of the *Drosophila* MKS complex in TZ formation we generated an *Mks1* mutation by imprecise P-element excision. This generated a 1.4 kb deletion that removed the N-terminal 474 aa of the MKS1 protein, including the start codon and part of the conserved B9 domain (Fig. 1B), a domain of unknown function often found in cilia- and/or flagella-associated proteins. We hereafter refer to flies homozygous for this mutation as *Mks1*^Δ*1*^ mutants.

We analysed the localisation of GFP fusions of the other TZ proteins by 3D-SIM in an *Mks1*^Δ*1*^ background. The TZ localisation of Cep290–GFP and Cby–GFP was not detectably perturbed, but B9D1–GFP, B9D2–GFP, TMEM216–GFP and Tectonic–GFP were no longer detectable at the TZ in *Mks1*^Δ*1*^ mutants (Fig. 1A). Interestingly, although CC2D2A–GFP was also no longer detectable at the TZ in *Mks1*^Δ*1*^ mutants, this protein seemed to re-localise to the BB walls (Fig. 1A). These findings strongly suggest that the recruitment of the entire MKS module of proteins to the TZ is dependent on MKS1, although a TZ that can recruit Cep290 and Cby is still formed in *Mks1*^Δ*1*^ mutants.

We also generated a mutation in the *B9d1* gene using CRISPR–Cas9 technology. This mutation generated a frame shift leading to the introduction of a premature stop codon and so presumably to the effective deletion of the C-terminal 164 aa of the B9D1 protein (Fig. 1C); we hereafter refer to flies homozygous for this mutation as *B9d1*^Δ*1*^ mutants. The TZ localisation of MKS1–GFP and B9D2–GFP were no longer detectable in *B9d1*^Δ*1*^ mutants (Fig. S2). As MKS1 is required for the localisation to the TZ of all the other MKS module proteins we have examined, the absence of MKS1 from the TZ of *B9d1*^Δ*1*^ mutants means that the other MKS module proteins are also probably absent from the TZ in *B9d1*^Δ*1*^ mutants. Thus, in agreement with previous reports (Bialas et al., 2009; Williams et al., 2008), MKS module proteins seem to be interdependent for their localisation to the TZ, and are not detectable at the TZ in *Mks1*^Δ*1*^ and *B9d1*^Δ*1*^ mutants.

### *Mks1*^Δ*1*^ and *B9d1*^Δ*1*^ mutants are viable, fertile and do not exhibit dramatic defects in sensory cilia function

**Fig. 2.**
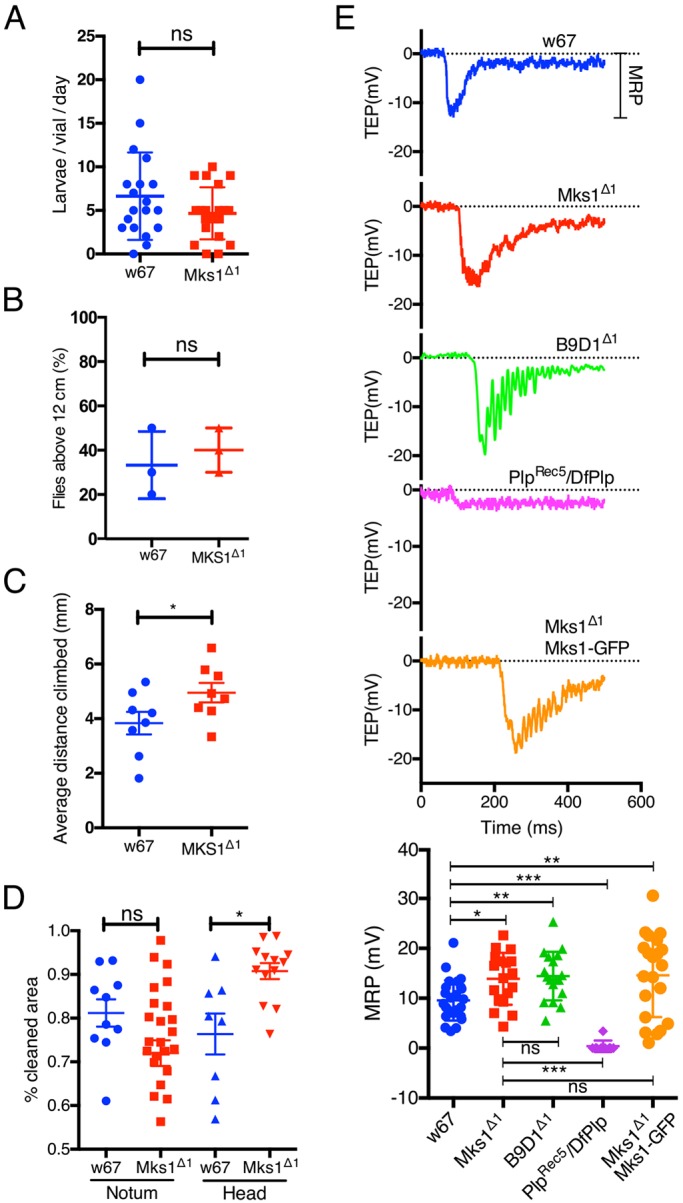
**MKS module mutants exhibit no dramatic behavioural defects.** (A) Graph quantifies male fertility of WT (*w^67^*) or *Mks1*^Δ*1*^ mutant males (see Materials and Methods). (B,C) Graphs quantify gravitaxis tests assessing the climbing reflex of WT (*w^67^*) or *Mks1*^Δ*1*^ mutant flies to climb to the top of a vial (B) and the speed at which flies climb (C). (D) Graph quantifies the grooming reflex test assessing the ability of WT (*w^67^*) or *Mks1*^Δ*1*^ mutant flies to clean dust from their notum or head. (E) Electrophysiology traces show typical examples of the trans-epithelial electron potential of individual mechanosensory bristles of the sensory notum cilia recorded from either WT, *Mks1*^Δ*1*^, *B9d1*^Δ*1*^ or (as a negative control) *Plp^Rec5/^DfPlp* flies. A trace from an *Mks1*^Δ*1*^ mutant rescued by the MKS1–GFP transgene is also shown. The graph (bottom panel) quantifies the average mechanical response potential derived from these traces for each genotype. Error bars represent the s.d. Significance was assessed using a one-way ANOVA; *P<0.01, **P<0.001, ***P<0.0001; ns, not significant.

To our surprise, *Mks1*^Δ*1*^ and *B9d1*^Δ*1*^ mutants were not noticeably uncoordinated. We have maintained homozygous stocks of these mutants in the laboratory for more than two years, indicating that they are both male and female fertile. This is in contrast to previously described mutations in the TZ protein encoding genes *Cep290* (Basiri et al., 2014), *Cby* (Enjolras et al., 2012) and *dila* (Ma and Jarman, 2011) that are uncoordinated and exhibit reduced male fertility. In quantitative fertility tests *Mks1*^Δ*1*^ mutant males were not significantly less fertile than WT controls, indicating that mutant sperm flagella were largely functional (Fig. 2A).

We next performed a series of assays to test sensory cilia function. We first tested the flies' geotaxis response (Hirsch and Tryon, 1956). When WT flies are knocked down to the bottom of a tube they quickly climb to the top, whereas flies with defective sensory cilia function are uncoordinated and cannot climb efficiently. There was no significant difference in the number of flies counted at the top of the vial 10 s after knock down between WT and *Mks1*^Δ*1*^ mutants (Fig. 2B), but we noticed that *Mks1*^Δ*1*^ flies seemed to climb faster than WT. We therefore repeated the assay, but measured the average distance climbed per vial after 7 s of knockdown. This confirmed that the *Mks1*^Δ*1*^ mutants climb slightly faster than WT (Fig. 2C). We next compared the dust grooming response of WT and *Mks1*^Δ*1*^ mutant flies, which is driven by the mechanical stimulation of external bristles by dust (Phillis et al., 1993). This revealed that *Mks1*^Δ*1*^ flies had a significantly higher cleaned area on their heads but no difference was observed in the notum (Fig. 2D). Taken together, these observations confirm that sensory cilia function in *Mks1*^Δ*1*^ mutants is not dramatically perturbed and that, if anything, mutant flies exhibit a slightly increased response to certain stimuli (see below), although such a subtle difference could simply result from genetic background differences.

### Mechanoreceptor potentials of *Mks1*^Δ*1*^ and *B9d1*^Δ*1*^ mutant sensory cilia are not dramatically perturbed

To more directly examine sensory cilium function in *Mks1*^Δ*1*^ mutants we measured the electrophysiological response of mechanosensory bristles in the notum. Each of these bristles is innervated by a peripheral sensory neuron that extends a cilium at the base of the bristle. The supporting cells that encapsulate the cilium generate a trans-epithelial electron potential (TEP) that can be detected by placing one electrode over the cut end of a bristle and inserting a reference electrode into the thorax (Kernan et al., 1994). Deflecting the bristle with a mechanical stimulus causes ion channels in the cilium membrane to open, allowing ions to flow from the support cells and generate a mechanical response potential (MRP). MRPs in *Mks1*^Δ*1*^ mutants and in *B9d1*^Δ*1*^ mutants did not differ dramatically from controls; in fact both mutants exhibited a slight, but statistically significant, increase in MRP amplitude (Fig. 2E). As a negative control we made similar recordings in *Pericentrin-like-protein* (*Plp*) mutants that have previously been shown to have severely reduced sensory cilia function (Martinez-Campos et al., 2004) (Fig. 2E). We also analysed a line expressing MKS1–GFP in the *Mks1*^Δ*1*^ mutant background and observed that this strain also had a slight increase in the MRP response (Fig. 2E), suggesting that the slight increase in sensitivity is either a result of genetic background differences between the WT and mutant strains, or is not efficiently rescued by the MKS1–GFP transgene. In either case, the electrophysiological response of the *Mks1*^Δ*1*^ and *B9d1*^Δ*1*^ mutant sensory cilia clearly falls within the normal range (Dubruille et al., 2002).

### *Mks1*^Δ*1*^ mutant spermatocyte axonemes are shortened, but mutant testes exhibit no dramatic defects

**Fig. 3.**
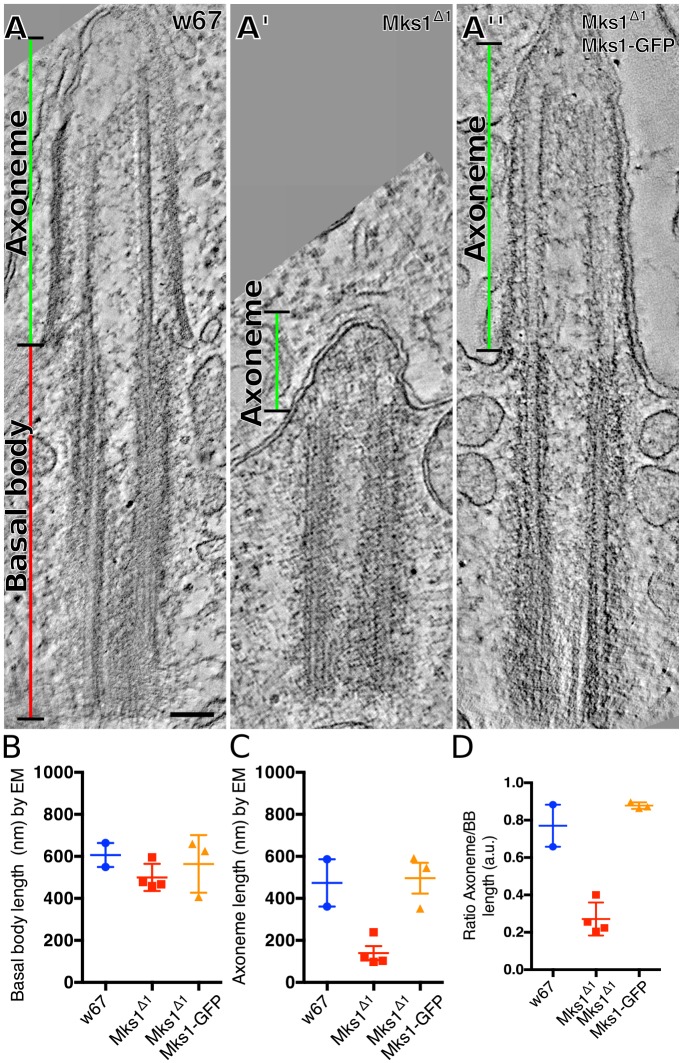
***Mks1*^Δ*1*^ mutant primary spermatocytes have short axonemes.** (A-A″) Micrographs show electron tomograms of primary spermatocyte cilia from WT (A), *Mks1*^Δ*1*^ mutant (A′) and *Mks1*^Δ*1*^ mutant rescued by MKS1–GFP (A″). (B-D) Graphs show the quantification of BB (B), axoneme (C) and the ratio of the axoneme/BB (D) lengths in WT (blue dots), *Mks1*^Δ*1*^ mutant (red squares) and *Mks1*^Δ*1*^ mutant rescued by MKS1–GFP (orange triangles) measured from EM tomograms. Error bars represent the s.d. Note that only 2–4 tomograms from each genotype contained the entire BB–axoneme complex, so we did not perform statistical analysis of significance. Scale bar: 100 nm.

To better understand why the lack of MKS module proteins seems to have only a very mild effect on cilia and flagella function, we examined cilia and flagella structure by EM tomography. We first examined testes, as MKS module proteins are most highly expressed in this tissue and mutations in *Cep290*, *Cby* and *dila* all exhibit severe defects in testes (Basiri et al., 2014; Enjolras et al., 2012; Ma and Jarman, 2011). An initial TEM examination of *Mks1*^Δ*1*^ mutant flagella in cross section revealed no obvious structural defects, consistent with our observations that mutant flies are male fertile (Fig. S3). However, we noticed that the short axonemes that are normally assembled in mature spermatocytes (A.D. Tates, PhD thesis, Rijksuniversiteit Leiden, The Netherlands, 1971) were dramatically shortened in mutant cells (Fig. 3A,C,D; Movies 1 and 2), whereas the length of the very long BBs at the base of these axonemes was not dramatically affected (Fig. 3B). Importantly, this short axoneme phenotype was rescued by the expression of the MKS1–GFP transgene (Fig. 3A,C,D; Movie 3). These findings strongly suggest that the proper assembly of the short spermatocyte axoneme is dependent on MKS module proteins.

### The localisation of the transmembrane proteins NompA and NompC is not detectably perturbed in *Mks1*^Δ*1*^ mutant sensory cilia

**Fig. 4.**
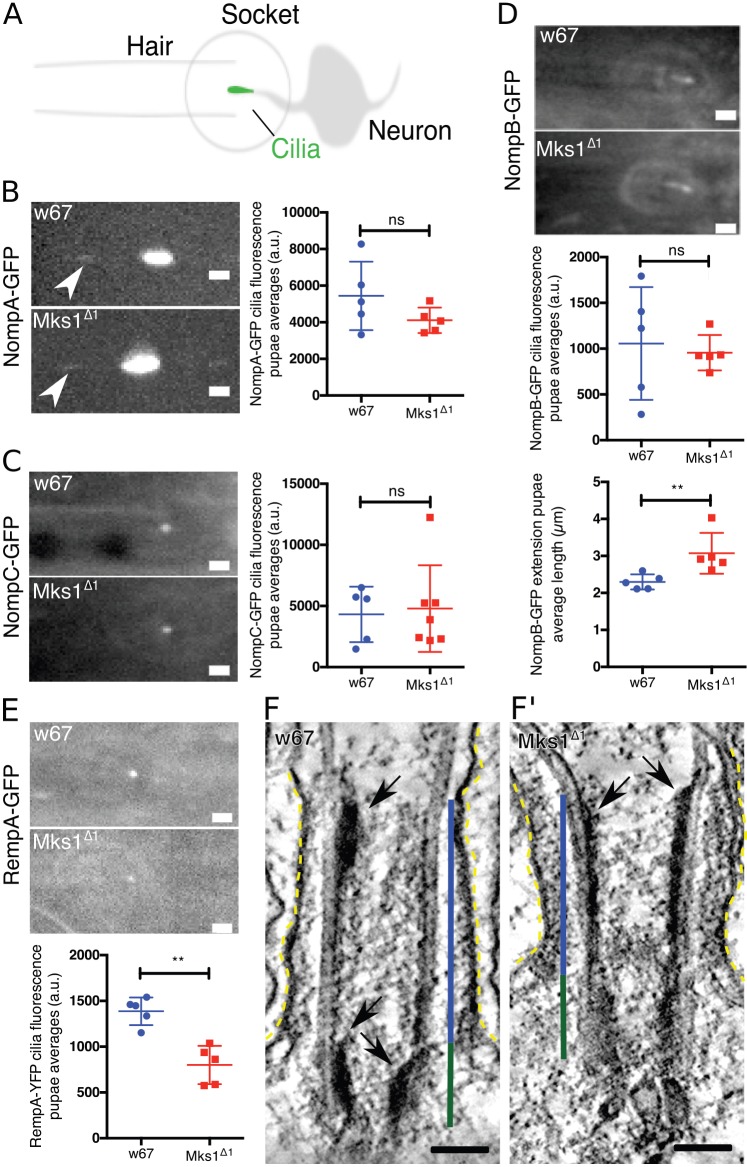
***Mks1^Δ1^* mutant sensory cilia seem to have subtle defects in the localisation of IFT proteins.** (A) A schematic illustration of the *Drosophila* notum hair socket and associated sensory cilia. (B-E) Micrographs and quantification of fluorescence intensity of the transmembrane proteins (B) NompA–GFP (which localises to the dendritic cap of the cilium), (C) NompC–GFP (a ciliary ion channel protein), (D) NompB–GFP (the *Drosophila* homolog of IFT88) and (E) RempA–GFP (the *Drosophila* homolog of IFT144) in WT and *Mks1*^Δ*1*^ mutant sensory cilia. The localisation of NompA–GFP and NompC–GFP was not detectably perturbed in *Mks1*^Δ*1*^ mutants, but significantly less RempA–GFP localised in *Mks1*^Δ*1*^ mutant cilia, whereas the average length of NompB–GFP staining (E, bottom panel) revealed that this was slightly longer in *Mks1*^Δ*1*^ mutants. NompA–GFP localised strongly to the base of the bristle, with a dimmer and elongated dot in the bristle hair (arrow). (F,F′) Micrographs show electron tomograms of the BB (dark green lines) and TZ (blue lines) in a WT (F) and an *Mks1*^Δ*1*^ mutant (F′) sensory cilium (ciliary membrane is highlighted by dotted yellow lines). Electron-dense particles similar to IFT trains (arrows) are visible on the BB microtubules facing the TZ lumen in WT. In *Mks1*^Δ*1*^ sensory cilia these particles seem to be dramatically extended. Error bars represent s.d. Significance was assessed using a two-tailed *t*-test; **P<0.001; ns, not significant. Scale bars: 2 µm in B–D, 100 nm in F,F′.

The slight increase in signalling response in the bristle hairs suggested a possible defect in protein composition in the ciliary membranes of *Mks1*^Δ*1*^ mutants. We therefore examined the distribution of the transmembrane proteins NompA and NompC. NompA is localised to the dendritic cap at the distal tip of the cilium and is essential for cilia function (Chung et al., 2001; Kernan et al., 1994), whereas NompC forms ion channels in the ciliary membrane that are enriched towards the distal tip of the cilium (Lee et al., 2010; Zhang et al., 2013). We looked at the notum of flies 72 h after pupae formation (APF) and found that in both WT and *Mks1*^Δ*1*^ mutant cells NompA–GFP localised strongly to the base of the bristle, with a dimmer and elongated dot in the bristle hair (Fig. 4A,B), whereas NompC–GFP localised to a single dot at the bristle base (Fig. 4C). This suggests that the distribution of ion channels in the ciliary membrane is not dramatically perturbed in *Mks1*^Δ*1*^ mutant cilia.

### Intraflagellar transport seems to be subtly perturbed in *Mks1*^Δ*1*^ mutant cilia

Previous studies have suggested that MKS proteins interact with intraflagellar transport (IFT) proteins in zebrafish cilia (Zhao and Malicki, 2011). To test this possibility more directly in flies, we examined the localisation of NompB–GFP – the IFT88 homolog responsible for anterograde transport (Han et al., 2003) – and of RempA–YFP – the IFT140 homolog that is part of the retrograde transport system (Lee et al., 2008) – at the base of the notum bristle. In WT cells, NompB–GFP localised as a line at the base of the cilium that was enriched at the proximal end of the axoneme, and this was also the case in *Mks1*^Δ*1*^ mutants, although the NompB–GFP signal was slightly, but significantly, extended (Fig. 4D). RempA–YFP localised as a dot at the base of the bristle hair in both WT and mutant cells, although its intensity was significantly reduced in mutants (Fig. 4E). These studies support the possibility that there are subtle defects in IFT in *Mks1*^Δ*1*^ mutant cilia.

IFT particles are often visible by EM as electron-dense ‘IFT trains’ located between the MTs and the ciliary membrane (Rogowski et al., 2013; Stepanek and Pigino, 2016; Vannuccini et al., 2016). Interestingly, in our EM analysis of WT cilia we could not find such particles localised between the B MTs and the ciliary membrane in the axoneme, but we instead found similar extended electron-dense particles localising along the A MTs and facing the internal lumen of the BB–TZ junction (arrows, Fig. 4F). These findings raise the intriguing possibility that IFT particles might assemble on the intraluminal surface of the BB in these cells. Supporting the idea that these particles might be IFT particles, we observed similar particles in *Mks1*^Δ*1*^ mutants at 72 h APF (Fig. 4F′) but these particles seemed to accumulate at the BB, forming a continuous sheet from the proximal lumen of the BB and into the TZ [137±32.5 nm (mean±s.d.) in nine particles out of three cilia for WT vs 184.8±70.1 nm in 11 particles out of three cilia for *Mks1^Δ1^*] – in accordance with the increase in length of the NompB–GFP signal in *Mks1*^Δ*1*^ mutant sensory cilia (Fig. 4D). We concluded that IFT is probably subtly perturbed in *Mks1*^Δ*1*^ mutant sensory cilia.

### *Mks1*^Δ*1*^ mutant cilia exhibit striking structural defects at 48 h APF, but are largely normal by 72 h APF

**Fig. 5.**
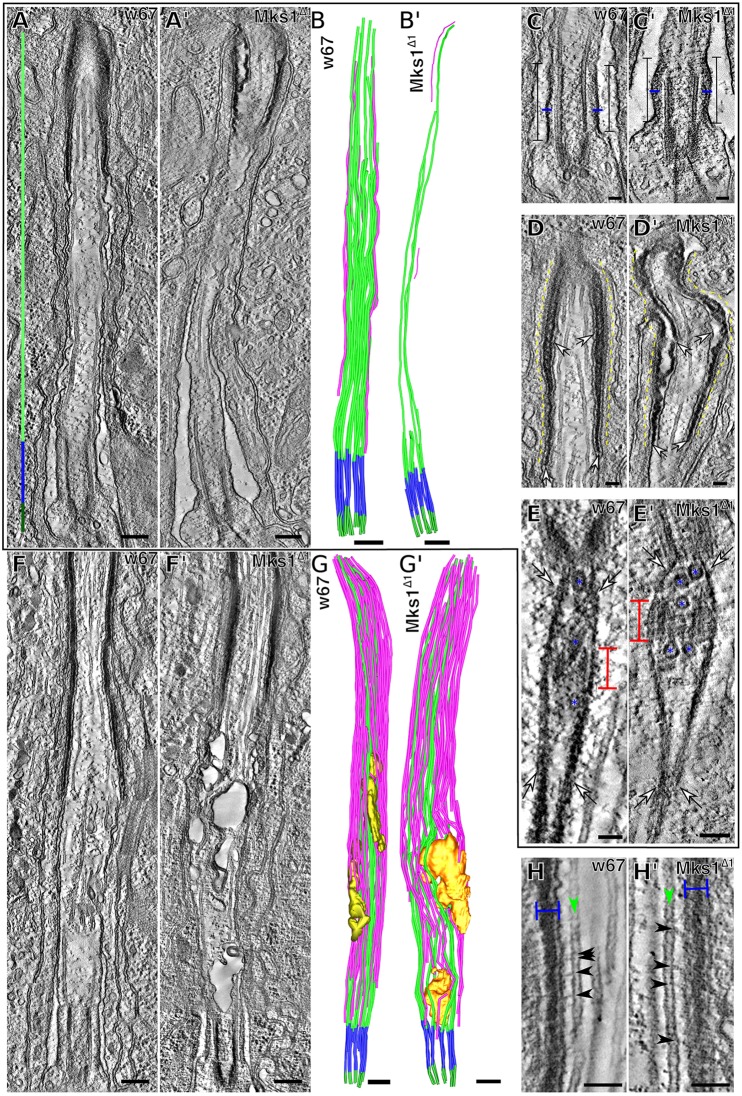
***Mks1*^Δ*1*^ mutant sensory cilia exhibit structural defects at 48 h APF, but are relatively normal by 72 h APF.** (A,A′) Micrographs show electron tomograms of the sensory cilium at 48 h APF in WT (A) and *Mks1^Δ1^* mutants (A′). (B,B′) Illustrations show the corresponding 3D models of A,A′, highlighting the BB MTs (dark green), the TZ MTs (blue) and axonemal MTs (light green). Axoneme MTs not directly attached to the BB are highlighted in magenta. (C,C′) Higher magnification electron tomograms of the TZ (square capped lines) in WT (C) and *Mks1^Δ1^* mutants (C′); the TZ is shorter in the mutant, and the electron-dense TZ membrane (blue lines) seems wider and more diffuse. (D,D′) At the tip of the WT axoneme there is a crystalline-like electron-dense structure (D, arrows) located between the ciliary membrane (adjacent to upper arrows) and the sheath cell membrane (highlighted by dotted yellow lines); this structure is present in the *Mks1^Δ1^* mutant cilia (D′) (lower arrows) but the region is less-well organised, and there are gaps between the ciliary and sheath cell membranes (upper arrows). (E,E′) Electron tomograms of the area just below the ciliary BB in WT (E) and *Mks1^Δ1^* mutants (E′) reveal the rootletin filaments (arrows) that originated from the BB, surround the daughter centriole (red capped lines) and extend into the neuronal cell body. Several vesicles (blue asterisks) are visible at the base of the BB and the daughter centriole in the space delimited by the rootlet. Analysis of this area revealed no obvious differences between WT and *Mks1^Δ1^* mutants. (F–G′) Electron tomograms (F,F′) and the corresponding 3D models (G,G′) of WT (F,G) and *Mks1^Δ1^* (F′,G′) sensory cilia at 72 h APF. The number of axonemal MTs increased from 48 h to 72 h, and mutant cilia appear more similar to WT, although an internal membranous structure can be discerned (gold) that is more extensive in *Mks1^Δ1^* mutant cilia. (H,H′) At 72 h APF, electron-dense bridges (black arrowheads) connecting the MTs (green arrowheads) and the ciliary inner membrane are visible in both WT (H) and *Mks1^∆1^* mutants (H′) towards the cilium tip where the electron-dense crystalline-like structure is present (blue capped line) and appears to be organised in a similar way in both WT and *Mks1^Δ1^* mutants. Scale bars: 500 nm in A, A′,B, B′,F,F′,G,G′; 100 nm in all other images.

We next used ET to compare the ultrastructure of the sensory cilia in the bristles of the notum in WT and *Mks1*^Δ*1*^ mutants at 48 h and 72 h APF (Fig. 5). At 48 h APF WT cilia (*n*=3) form relatively straight cilia that extend towards the PM of the hair cell (Fig. 5A,B; Movie 4). At their base, these cilia have a BB (BB MTs shown in dark green) that extends MT doublets into the TZ (blue) and then into the ciliary membrane (light green). The TZ is discernible as a straightening (black bar, Fig. 5C) and electron-dense thickening (blue bar, Fig. 5C) of the membrane, similar to that visualised in spermatocyte cilia (Fig. 3A). The ciliary MTs doublets extend for ∼2/3 of the cilia length and then continue as singlets of the A MTs; most of these A MTs terminate close to the tip of the cilium. Some MTs that were not connected to the BB were also found in the axoneme in close apposition to the ciliary membrane (purple) and an electron-dense crystalline structure surrounded the top ∼1/3 of the axoneme, apparently linking the outer ciliary membrane with the adjacent sheath cell membrane (arrows, Fig. 5D). At the base of the cilium, a daughter centriole was positioned just below the BB and was surrounded by ciliary rootlets (Chen et al., 2015; Styczynska-Soczka and Jarman, 2015) emanating from the distal end of the BB (Fig. 5D) where vesicles could also be observed (blue asterisks, Fig. 5E).

**Fig. 6.**
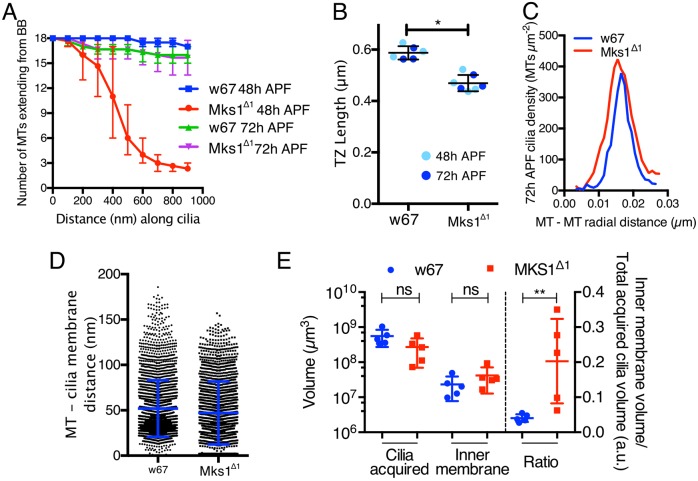
**A comparison of some of the features of WT and *Mks1*^Δ*1*^ sensory cilia.** (A) Graph shows the quantification of the number of MTs originating from the BB that elongate into the cilium in WT and *Mks1*^Δ*1*^ sensory cilia at 48 h and 72 h APF. These data are obtained from three cilia for each genotype. (B) Graph shows the quantification of TZ length (measured from EM tomograms) in WT and *Mks1*^Δ*1*^ sensory cilia at 48 h and 72 h APF. (C) Graph shows that average spacing between the ciliary MTs is ∼16 nm in both WT and *Mks1*^Δ*1*^ mutant cilia at 72 h APF. (D) Graph shows the quantification of distance between the cilia MTs and the ciliary membrane in WT and *Mks1*^Δ*1*^ sensory cilia at 72 h APF. (E) Graph shows the quantification of the total ciliary volume acquired, the volume of the cilia internal membrane acquired and the ratio of the two (all data from EM tomograms of 72 h APF cilia). The *Mks1*^Δ*1*^ mutant cilia appear slightly smaller and have a slightly larger volume of internal membranes, but these differences are not significant (perhaps owing to the low *n*-numbers as we could only reconstruct a relatively small number of cilia); the ratio of these parameters, however, reveals a clear difference between the mutant and WT. Error bars represent range in A and s.d. in all other graphs. Significance was assessed with a two-tailed *t*-test; *P<0.01, **P<0.001; ns, not significant.

*Mks1*^Δ*1*^ mutant cilia (*n*=3) were generally thinner than WT cilia and the ciliary MTs were much more sparse (Fig. 5A′,B′; Movie 5). This was because most BB MTs terminated just above the TZ with only ∼1–3 MTs extending into the cilium proper, compared with the 17–18 MTs observed in this region of WT axonemes (Fig. 5A,B; Fig. 6A). The crystalline-like structure at the tip of the axoneme was also more disorganised and the connection between the crystalline structure and the ciliary and sheath membranes contained several gaps (arrows, Fig. 5E′). The TZ was also noticeably shorter (Fig. 5A′,B′; Fig. 6B) and the electron-dense region of the TZ membrane was more diffuse than in WT (blue bar, Fig. 5C′). The arrangement of the centriole pair and ciliary rootlets at the base of the cilium, however, seemed largely unperturbed (Fig. 5D′). Thus, the ultrastructural organization of the 48 h APF cilia is strongly disrupted in *Mks1*^Δ*1*^ mutant sensory neurons. Importantly, this is not owing to the *Mks1*^Δ*1*^ mutant developing more slowly than WT, as the time from pupation to eclosion was similar in WT and mutant [4.6±0.1 days (mean±s.d.) at 25°C, *P*>0.999].

At 72 h APF the overall organisation of the WT cilium (Fig. 5E,F; Movie 6) was similar to that seen at 48 h APF, although the volume of the cilium had increased and the cilium contained many more MTs that ran along the main axis of the cilium but that were not directly connected to the BB. Most of these MTs were in close contact with the ciliary membrane and small links between the MTs and the membranes were visible (black arrows, Fig. 5H), similar to the membrane–microtubule connectors previously observed in chordotonal cilia (Young, 1973). These MTs had a radial separation of ∼16 nm (Fig. 6C) and were on average ∼51 nm (median of ∼41 nm) from the ciliary membrane (Fig. 6D). In addition, the amount of internal membrane present within the 72 h APF cilium also increased (gold, Fig. 5F,G; Fig. 6E), and this extra membrane seemed to originate from invaginations of the cell membrane into the lumen of the cilium (arrow, Fig. S4). The inner volume of this membrane was therefore in contact with the extracellular cytoplasm. The membrane at these regions was very electron­-dense, suggesting the presence of high levels of protein material and/or saturated lipids (Fig. 5E).

Interestingly, the 72 h APF *Mks1*^Δ*1*^ mutant cilia had a MT organization that was much more similar to WT than seen at 48 h APF, and many of the BB MTs now extended to the tip of the cilium and large numbers of non-ciliary MTs were also present aligned along the long axis of the cilium (Fig. 5F′,G′; Movie 7). We noticed, however, that there was a larger volume of inner membrane within *Mks1^Δ1^* cilia compared with WT cilia (gold, Fig. 5F′,G′; Fig. 6E), and the ratio of cilium volume to inner membrane volume seemed significantly increased in *Mks1^Δ1^* mutants (Fig. 6E).

## DISCUSSION

The TZ is essential for proper cilia function (Czarnecki and Shah, 2012; Reiter et al., 2012), and recent studies have suggested that an MKS and an NPHP module work partly redundantly with a Cep290 module to establish the TZ (Basiri et al., 2014; Schouteden et al., 2015; Williams et al., 2008, 2011). The NPHP module seems to have arisen later in evolution and to be largely absent in *Drosophila*, but six potential members of the MKS module have been identified in flies (Barker et al., 2014; Basiri et al., 2014). We show here that mutations that disrupt the function of either of two members of the MKS module, MKS1 or B9D1, strongly disrupt the TZ-localisation of the other MKS module components, but do not detectably disrupt the TZ localisation of Cep290 or the TZ component Cby, strongly supporting the idea that the MKS module proteins form a functional unit in flies. Despite the apparent lack of a functional MKS module in *Mks1*^Δ*1*^ or *B9d1*^Δ*1*^ mutants, these flies exhibit only subtle cilia and flagella defects, and mutant flies are viable and fertile.

Although cilia function is only mildly perturbed in *Mks1*^Δ*1*^ and *B9d1*^Δ*1*^ mutants, the defects we do observe are potentially informative. The MKS module proteins are all highly expressed in testes and two types of cilia are formed in this tissue. Initially, short axonemes extend from the long centrioles found in mature spermatocytes, and these centrioles will later form the BB of the sperm flagella. In *Mks1*^Δ*1*^ mutants the short spermatocyte axonemes are dramatically reduced in length, although Cep290 and Cby are both localised normally at the base of the axoneme. The function of these short spermatocyte cilia is unknown, but our findings demonstrate that the axonemes of these cilia can be dramatically shortened without any obvious effect on the fly. In particular, the subsequent formation of the much longer sperm flagella is not detectably perturbed and mutant males seem to exhibit normal levels of fertility. This is in contrast to *Cep290* mutants that also have very short spermatocyte cilia (that seem to lack axonemal MTs), but subsequently exhibit dramatic defects in the formation of the sperm flagella axoneme (Basiri et al., 2014). It therefore remains unclear why MKS module proteins are so highly expressed in testes. We suspect that these proteins must contribute to cilia and flagella function in the testes, but that this function is not apparent in the assays we have used here. Nevertheless, it is clear that Cep290 can organise a sufficiently functional TZ in fly testes in the apparent absence of the MKS module, but MKS module proteins cannot do the same in the absence of Cep290 (Basiri et al., 2014).

*Cep290* mutant flies are also severely uncoordinated because of defects in their sensory cilia (Basiri et al., 2014), whereas *Mks1*^Δ*1*^ mutants are not noticeably uncoordinated and perform at least as well as WT flies in various assays that assess cilia function. Indeed, mutant flies seem to climb slightly faster than WT flies, and the mechanosensory cilia that attach to the bristles on the notum are, if anything, slightly more sensitive than WT. Although the distribution of the cilia membrane ion channel NompC protein was not detectably perturbed in *Mks1*^Δ*1*^ mutant sensory cilia, the ratio of inner membrane volume to total cilium volume seemed increased in 72 h APF mutant sensory cilia. These internal membranes seem to be formed from invaginations of the plasma membrane, so the increase in membrane volume could lead to an increase in ion flow upon membrane channel opening. It is unclear, however, how this increase in inner membrane comes about. One possibility is that the lack of ciliary MTs and the looser organisation of the ciliary membrane we observe at earlier stages of development underlie the eventual enlargement of the inner membrane structures in *Mks1^*Δ*1^* cilia.

The localisation and distribution of the IFT components RempA and NompB were also subtly perturbed in *Mks1*^Δ*1*^ mutant cilia, indicating a potential defect in IFT, as has been suggested previously for MKS module mutants (Zhao and Malicki, 2011). Moreover, in WT sensory cilia our EM analysis identified extended electron-dense ‘particles’ on the inner lumen of the BB that seemed to be similar to the electron-dense ‘trains’ of IFT particles previously described in axonemes and that localise between the axonemal MTs and the plasma membrane. In *Mks1*^Δ*1*^ mutants these particles seemed to accumulate in the lumen of the BB. Clearly, more work will be required to determine the identity and composition of these structures, but these observations raise the intriguing possibility that IFT particles can assemble on the inner lumen of the BB wall in these cilia. Supporting this idea, this accumulation is in agreement with our results showing that NompB–GFP localisation is extended in *Mks1*^Δ*1*^, and with previous work that also show an accumulation of NompB in RempA mutants in the chordotonal organ cilia (Lee et al., 2008). Taken together, these results suggest that *Mks1* initially affects the localisation of RempA, which leads to the abnormal accumulation of NompB.

Although sensory cilia structure was only moderately perturbed in *Mks1*^Δ*1*^ mutant pupae at 72 h APF, cilia structure was more profoundly disrupted earlier in development at 48 h APF. Most strikingly, the axoneme in these developing pupae had severely disrupted MTs, with very few of the BB MTs extending into the axoneme. Thus, the MKS module seems to have an important function in initially establishing proper cilia structure in flies but, given enough developmental time, a largely functional cilium can be established and maintained in the absence of these proteins. More work will be necessary to explain the mechanistic basis of these early defects in cilia structure, but as an IFT-B component can regulate ciliary MT growth by negatively regulating the microtubule stabilising protein MAP4 (Bizet et al., 2015), and *Mks1*^Δ*1*^ seems to lead to an accumulation of the fly IFT-B component NompB, a similar process could be occurring in flies.

Our results, together with previous studies (Basiri et al., 2014; Enjolras et al., 2012; Ma and Jarman, 2011) suggest a model of sequential assembly of TZ assembly in flies. BB docking to the PM triggers the recruitment of the TZ components, which rely on Cep290 for correct localisation. Cep290 is essential for proper TZ function in flies and for the correct migration of the TZ along the axoneme in growing spermatids (Basiri et al., 2014). Cby plays an important role in these processes, but might not be essential as *Cby* mutants are uncoordinated, show reduced mechanosensation, and reduced, but not abolished, male fertility – indicating the existence of at least a semi-functional cilium and/or flagullum. In contrast, although the MKS complex is required for the assembly of the short axoneme in spermatocytes, cilia and flagella function in adult flies seems only mildly perturbed in the absence of the complex. Previous studies in mice and worms have also indicated that MKS mutants exhibit relatively mild cilia defects (Dawe et al., 2007; Slaats et al., 2015; Tammachote et al., 2009; Weatherbee et al., 2009; Williams et al., 2008, 2011), but this is often attributed to the MKS module acting redundantly with the NPHP module (Williams et al., 2008, 2011; Yee et al., 2015), something that seems unlikely in flies as they lack most of the core NPHP module proteins (Barker et al., 2014; Basiri et al., 2014).

An intriguing possibility is that the MKS module in flies acts partially redundantly with Cby, and Cby mutants exhibit a similar NompB accumulation at the TZ as MKS mutants (Enjolras et al., 2012). Alternatively, the MKS module could also act partially redundantly with proteins of the Bardet–Beidel syndrome complex (BBSome) that is required for cilium function, vesicle trafficking and IFT in several systems (Jin et al., 2010; Wei et al., 2012; Williams et al., 2014). Perturbing BBS function modifies the phenotype of Cep290, MKS or NPHP mutations in other systems (Barbelanne et al., 2015; Yee et al., 2015; Zhang et al., 2014), but the BBS complex remains uncharacterised in flies.

## MATERIALS AND METHODS

### Fly stocks

*w^67^* was used as a control in all experiments. *Mks1*^Δ*1*^ (this study) was generated by imprecise excision of a p-element [p{Epgy2}CG2556EY2042 (Bloomington); Rubin and Spradling, 1982] 581 bp upstream of *Drosophila* *Mks1*. *B9d1*^Δ*1*^ (this study) was generated by CRISPR–Cas9. Three guide RNAs (gRNAs: GAAAGGTGGCCGAGACTATTTGG, CATCGTGGGCCAAATAGTCTCGG and CATCAGTCTCCCGGGCAACGAGG) were cloned into the pCFD3-dU6:3gRNA plasmid (Port et al., 2014) and injected into *Drosophila* embryos expressing the *Cas9* gene under the control of the *Nanos* promoter (Port et al., 2014). *Plp^Rec5^* was published previously (Martinez-Campos et al., 2004). Mks1–GFP, B9D1–GFP, B9D2–GFP, TMEM216–GFP, Tectonic–GFP (all this study) are expressed from the *Ubiquitin* promoter, which drives moderate expression in all tissues (Lee et al., 1988). CC2D2A–GFP (this study) is driven by the endogenous promoter by including a ∼2 kb upstream region in the transgene. Transgenes were cloned into Gateway (Invitrogen) vectors as described before (Basto et al., 2006) labelled with *w^+^*. The following transgenic lines were published previously and were kind gifts: Cby–GFP (B. Durand; Enjolras et al., 2012), Cep290–GFP (T. Avidor-Reiss; Basiri et al., 2014), NompA–GFP (R. Stanewsky; Chung et al., 2001), NompB–GFP (Maurice Kernan; Han et al., 2003), NompC–GFP (Y. Nung; Yan et al., 2013), NompC-Gal 4 (Y. Nung; Yan et al., 2013), RempA–YFP (A. Jarman; Lee et al., 2008).

### Behaviour assays

To quantify the pupal development, *Mks1*^Δ*1*^ or *w^67^* white pupae were marked with date and time and kept at 25°C. Hatched pupae were counted every morning and evening. Data is presented as the average of three technical repeats of at least five pupae per repeat. For male fertility, single *Mks1*^Δ*1*^ or *w^67^* flies were crossed to three *w^67^* females. Hatched embryos were counted every morning and evening for 6 days. Each of the 25 biological replicates is presented as the average number of hatched embryos per day. The standard climbing assay was adapted from Ma and Jarman (2011). Briefly, 15 adult flies at 1–3 days old were knocked to the bottom of a cylinder and the number of flies above 12 cm counted after 10 s. Data are the averages of three technical replicates. To measure the distance climbed by individual flies, five technical replicates of 10 adult flies at 1–3 days old were filmed being knocked to the bottom and crawling up the sides of the cylinder. The experimental time was reduced to 7 s to prevent flies reaching the top of the cylinder. The distance climbed per fly was measured in Fiji (Schindelin et al., 2012). For the grooming assay, the average of 10 flies at 1–3 days old per time point were covered in Reactive Yellow 86 dust (Organic Dyestuffs Corporation) (Seeds et al., 2014). The flies were then left to groom themselves for 30 or 90 min before imaging the head and notum on a dissecting microscope equipped with a Digital Sight camera (Nikon). A polygon ROI of the head or notum was used to measure their area. Next, the colour threshold was set to the Reactive Yellow 86 dust to select the areas covered in the dust. The area of these parts within the original ROI was also measured and the ratio was determined.

### Electrophysiology

Bristle recordings were collected from individual males at 2–4 days old as described in (Kernan et al., 1994). Humeral and notopleural bristles were cut along their midpoint and a tungsten wire reference electrode was inserted into the thorax. A glass capillary electrode containing 121 mM K^+^, 9 mM Na^+^, 0.5 mM Ca^2+^, 4 mM Mg^2+^, 35 mM glucose, and 5 mM HEPES, pH 7.1, was placed over the cut bristle to record MRPs. MRPs were evoked by 30 μm deflections of the electrode by software-controlled movement of a PatchStar micromanipulator (Scientifica). Analogue signals were acquired through a MultiClamp-700B amplifier and digitised with an Axon Digidata 1550A A/D board. Data were collected in Clampfit 10.5 (Molecular Devices) and MRP amplitudes were measured offline. Mean amplitudes from each genotype were then compared using a one-way ANOVA with Tukey's multiple comparisons test in Prism 6 (GraphPad).

### Fluorescence microscopy

3D-SIM of testis was performed as described previously (Roque et al., 2012). Live imaging was performed in a Nikon Eclipse TE200-E spinning disk confocal system, equipped with an EM-CCD Andor iXon+ camera, controlled by the Andor IQ2 software. Pupae were prepared for imaging as previously described (Jauffred and Bellaiche, 2012).

### Image analysis

The barrel-shaped diameter of GFP-tagged TZ proteins was calculated by fitting a double Gaussian model to an intensity profile line perpendicular to the longitudinal axis of the axoneme, and measuring the distance between peaks. The same fitting was used to calculate the full width half maximum (FWHM) value for each peak and the average was used as the thickness of the tube wall. For Cep290–GFP, a single Gaussian curve was fitted and the FWHM calculated. Profile curves were plotted with Fiji (Schindelin et al., 2012) and fitting done with Prism 6 software (GraphPad). Distributions were tested for Gaussian distributions by the D'Agostino and Pearson omnibus test. Significance between distributions was tested by an unpaired *t*-test for Gaussian distributions and the Mann–Whitney test for non-Gaussian distribution. Composed images of ETs and the localisation maps of TZ proteins obtained by 3D-SIM were created by scaling the Asl staining quantifications to the EM micrograph BB wall and applying the same scaling to the remaining quantifications. The intensities of NompA–GFP, NompB–GFP, NompC–GFP and RempA–GFP were measured in Fiji by measuring the mean grey value of a ROI surrounding the signal in a sum projection and subtracting the adjacent background signal. The length of the NompB–GFP signal was measured in IMOD (Kremer et al., 1996) using open contours.

### Electron tomography

Testis samples were prepared as previously reported (Roque et al., 2012). This protocol was modified to prepare pupal samples for ET. Pupae were removed from their case at 48 or 72 h APF and placed into 2.5–4% glutaraldehyde–paraformaldehyde in 0.2 M PIPES buffer (fixative solution) and heptane for 1–2 h at room temperature. The pupae were transfer to 0.2 M PIPES and dissected with a pair of tungsten needles. The abdomen, head and cuticle were removed. The dissected samples were placed in fixative solution at 4°C overnight. Samples were washed three times for 15–30 m in 0.2 M PIPES. En-bloc staining was performed in 1% OsO_4_ in milliQ H_2_O for 90 m at room temperature, followed by five washes in milliQ H_2_O for 5 m each. Secondary fixation was performed in 1% uranyl acetate solution in milliQ water overnight followed by three washes in milliQ H_2_O. Dehydration was performed in a series of 30%, 50%, 75% and 100% EtOH for 45–60 m each, followed by 100% acetate for 45 min. Embedding was performed in a series of 25% (2 h), 50% (2 h) and 75% Agar100 in acetone (overnight), and 100% acetone overnight. The samples were polymerised at 60°C for 24 h. Tilt-series were acquired with SerialEM (Mastronarde, 2005) in a Tecnai T20 at 120 Kv and tomograms reconstructed and modelled with iMOD (Kremer et al., 1996).

## References

[JCS194621C1] Barbelanne M., Hossain D., Chan D. P., Peränen J. and Tsang W. Y. (2015). Nephrocystin proteins NPHP5 and Cep290 regulate BBSome integrity, ciliary trafficking and cargo delivery. *Hum. Mol. Genet.* 24, 2185-2200. 10.1093/hmg/ddu738 25552655 PMC4380068

[JCS194621C2] Barker A. R., Renzaglia K. S., Fry K. and Dawe H. R. (2014). Bioinformatic analysis of ciliary transition zone proteins reveals insights into the evolution of ciliopathy networks. *BMC Genomics* 15, 531 10.1186/1471-2164-15-531 24969356 PMC4092220

[JCS194621C3] Basiri M. L., Ha A., Chadha A., Clark N. M., Polyanovsky A., Cook B. and Avidor-Reiss T. (2014). A migrating ciliary gate compartmentalizes the site of axoneme assembly in Drosophila spermatids. *Curr. Biol.* 24, 2622-2631. 10.1016/j.cub.2014.09.047 25447994 PMC4254545

[JCS194621C4] Basten S. G. and Giles R. H. (2013). Functional aspects of primary cilia in signaling, cell cycle and tumorigenesis. *Cilia* 2, 6 10.1186/2046-2530-2-6 23628112 PMC3662159

[JCS194621C5] Basto R., Lau J., Vinogradova T., Gardiol A., Woods C. G., Khodjakov A. and Raff J. W. (2006). Flies without centrioles. *Cell* 125, 1375-1386. 10.1016/j.cell.2006.05.025 16814722

[JCS194621C6] Berbari N. F., O'Connor A. K., Haycraft C. J. and Yoder B. K. (2009). The primary cilium as a complex signaling center. *Curr. Biol.* 19, R526-R535. 10.1016/j.cub.2009.05.025 19602418 PMC2814769

[JCS194621C7] Bialas N. J., Inglis P. N., Li C., Robinson J. F., Parker J. D. K., Healey M. P., Davis E. E., Inglis C. D., Toivonen T., Cottell D. C. et al. (2009). Functional interactions between the ciliopathy-associated Meckel syndrome 1 (MKS1) protein and two novel MKS1-related (MKSR) proteins. *J. Cell Sci.* 122, 611-624. 10.1242/jcs.028621 19208769 PMC2720918

[JCS194621C8] Bizet A. A., Becker-Heck A., Ryan R., Weber K., Filhol E., Krug P., Halbritter J., Delous M., Lasbennes M.-C., Linghu B. et al. (2015). Mutations in TRAF3IP1/IFT54 reveal a new role for IFT proteins in microtubule stabilization. *Nat. Commun.* 6, 8666 10.1038/ncomms9666 26487268 PMC4617596

[JCS194621C9] Chen J. V., Kao L.-R., Jana S. C., Sivan-Loukianova E., Mendonça S., Cabrera O. A., Singh P., Cabernard C., Eberl D. F., Bettencourt-Dias M. et al. (2015). Rootletin organizes the ciliary rootlet to achieve neuron sensory function in Drosophila. *J. Cell Biol.* 211, 435-453. 10.1083/jcb.201502032 26483560 PMC4621839

[JCS194621C10] Chih B., Liu P., Chinn Y., Chalouni C., Komuves L. G., Hass P. E., Sandoval W. and Peterson A. S. (2011). A ciliopathy complex at the transition zone protects the cilia as a privileged membrane domain. *Nat. Cell Biol.* 14, 61-72. 10.1038/ncb2410 22179047

[JCS194621C11] Chung Y. D., Zhu J., Han Y.-G. and Kernan M. J. (2001). nompA encodes a PNS-specific, ZP domain protein required to connect mechanosensory dendrites to sensory structures. *Neuron* 29, 415-428. 10.1016/S0896-6273(01)00215-X 11239432

[JCS194621C12] Czarnecki P. G. and Shah J. V. (2012). The ciliary transition zone: from morphology and molecules to medicine. *Trends Cell Biol.* 22, 201-210. 10.1016/j.tcb.2012.02.001 22401885 PMC3331593

[JCS194621C13] Davis E. E. and Katsanis N. (2012). The ciliopathies: a transitional model into systems biology of human genetic disease. *Curr. Opin. Genet. Dev.* 22, 290-303. 10.1016/j.gde.2012.04.006 22632799 PMC3509787

[JCS194621C14] Dawe H. R., Smith U. M., Cullinane A. R., Gerrelli D., Cox P., Badano J. L., Blair-Reid S., Sriram N., Katsanis N., Attie-Bitach T. et al. (2007). The Meckel-Gruber Syndrome proteins MKS1 and meckelin interact and are required for primary cilium formation. *Hum. Mol. Genet.* 16, 173-186. 10.1093/hmg/ddl459 17185389

[JCS194621C111] Dubruille R., Laurençon A., Vandaele C., Shishido E., Coulon-Bublex M., Swoboda P., Couble P., Kernan M. and Durand B. (2002). Drosophila regulatory factor X is necessary for ciliated sensory neuron differentiation. *Development* 129, 5487-5498. 10.1242/dev.00148 12403718

[JCS194621C15] Enjolras C., Thomas J., Chhin B., Cortier E., Duteyrat J.-L., Soulavie F., Kernan M. J., Laurençon A. and Durand B. (2012). Drosophila chibby is required for basal body formation and ciliogenesis but not for Wg signaling. *J. Cell Biol.* 197, 313-325. 10.1083/jcb.201109148 22508513 PMC3328381

[JCS194621C16] Franz A., Roque H., Saurya S., Dobbelaere J. and Raff J. W. (2013). CP110 exhibits novel regulatory activities during centriole assembly in Drosophila. *J. Cell Biol.* 203, 785-799. 10.1083/jcb.201305109 24297749 PMC3857486

[JCS194621C17] Garcia-Gonzalo F. R. and Reiter J. F. (2012). Scoring a backstage pass: mechanisms of ciliogenesis and ciliary access. *J. Cell Biol.* 197, 697-709. 10.1083/jcb.201111146 22689651 PMC3373398

[JCS194621C18] Garcia-Gonzalo F. R., Corbit K. C., Sirerol-Piquer M. S., Ramaswami G., Otto E. A., Noriega T. R., Seol A. D., Robinson J. F., Bennett C. L., Josifova D. J. et al. (2011). A transition zone complex regulates mammalian ciliogenesis and ciliary membrane composition. *Nat. Genet.* 43, 776-784. 10.1038/ng.891 21725307 PMC3145011

[JCS194621C19] Han Y.-G., Kwok B. H. and Kernan M. J. (2003). Intraflagellar transport is required in Drosophila to differentiate sensory cilia but not sperm. *Curr. Biol.* 13, 1679-1686. 10.1016/j.cub.2003.08.034 14521833

[JCS194621C20] Hirsch J. and Tryon R. C. (1956). Mass screening and reliable individual measurement in the experimental behavior genetics of lower organisms. *Psychol. Bull.* 53, 402-410. 10.1037/h0040715 13359596

[JCS194621C21] Hsiao Y.-C., Tuz K. and Ferland R. J. (2012). Trafficking in and to the primary cilium. *Cilia* 1, 4 10.1186/2046-2530-1-4 23351793 PMC3541539

[JCS194621C22] Hu Q. and Nelson W. J. (2011). Ciliary diffusion barrier: the gatekeeper for the primary cilium compartment. *Cytoskeleton* 68, 313-324. 10.1002/cm.20514 21634025 PMC3143192

[JCS194621C23] Ishikawa H. and Marshall W. F. (2011). Ciliogenesis: building the cell's antenna. *Nat. Rev. Mol. Cell Biol.* 12, 222-234. 10.1038/nrm3085 21427764

[JCS194621C112] Jauffred B. and Bellaiche Y. (2012). Analyzing frizzled signaling using fixed and live imaging of the asymmetric cell division of the Drosophila sensory organ precursor cell. *Methods Mol. Biol.* 839, 19-25. 10.1007/978-1-61779-510-7_2 22218889

[JCS194621C24] Jensen V. L., Li C., Bowie R. V., Clarke L., Mohan S., Blacque O. E. and Leroux M. R. (2015). Formation of the transition zone by Mks5/Rpgrip1L establishes a ciliary zone of exclusion (CIZE) that compartmentalises ciliary signalling proteins and controls PIP2 ciliary abundance. *EMBO J.* 34, 2537-2556. 10.15252/embj.201488044 26392567 PMC4609185

[JCS194621C25] Jin H., White S. R., Shida T., Schulz S., Aguiar M., Gygi S. P., Bazan J. F. and Nachury M. V. (2010). The conserved Bardet-Biedl syndrome proteins assemble a coat that traffics membrane proteins to cilia. *Cell* 141, 1208-1219. 10.1016/j.cell.2010.05.015 20603001 PMC2898735

[JCS194621C26] Kernan M., Cowan D. and Zuker C. (1994). Genetic dissection of mechanosensory transduction: mechanoreception-defective mutations of drosophila. *Neuron* 12, 1195-1206. 10.1016/0896-6273(94)90437-5 8011334

[JCS194621C27] Kremer J. R., Mastronarde D. N. and McIntosh J. R. (1996). Computer visualization of three-dimensional image data using IMOD. *J. Struct. Biol.* 116, 71-76. 10.1006/jsbi.1996.0013 8742726

[JCS194621C28] Lee H. S., Simon J. A. and Lis J. T. (1988). Structure and expression of ubiquitin genes of Drosophila melanogaster. *Mol. Cell. Biol.* 8, 4727-4735. 10.1128/MCB.8.11.4727 2463465 PMC365564

[JCS194621C29] Lee E., Sivan-Loukianova E., Eberl D. F. and Kernan M. J. (2008). An IFT-A protein is required to delimit functionally distinct zones in mechanosensory cilia. *Curr. Biol.* 18, 1899-1906. 10.1016/j.cub.2008.11.020 19097904 PMC2615538

[JCS194621C30] Lee J., Moon S., Cha Y. and Chung Y. D. (2010). Drosophila TRPN(=NOMPC) channel localizes to the distal end of mechanosensory cilia. *PLoS ONE* 5, e11012 10.1371/journal.pone.0011012 20543979 PMC2882365

[JCS194621C31] Lee Y. L., Sante J., Comerci C. J., Cyge B., Menezes L. F., Li F.-Q., Germino G. G., Moerner W. E., Takemaru K.-I. and Stearns T. (2014). Cby1 promotes Ahi1 recruitment to a ring-shaped domain at the centriole-cilium interface and facilitates proper cilium formation and function. *Mol. Biol. Cell.* 25, 2919-2933. 10.1091/mbc.E14-02-0735 25103236 PMC4230582

[JCS194621C32] Li C., Jensen V. L., Park K., Kennedy J., Garcia-Gonzalo F. R., Romani M., De Mori R., Bruel A.-L., Gaillard D., Doray B. et al. (2016). MKS5 and CEP290 dependent assembly pathway of the ciliary transition zone. *PLoS Biol.* 14, e1002416 10.1371/journal.pbio.1002416 26982032 PMC4794247

[JCS194621C33] Ma L. and Jarman A. P. (2011). Dilatory is a Drosophila protein related to AZI1 (CEP131) that is located at the ciliary base and required for cilium formation. *J. Cell Sci.* 124, 2622-2630. 10.1242/jcs.084798 21750193 PMC3138703

[JCS194621C34] Martinez-Campos M., Basto R., Baker J., Kernan M. and Raff J. W. (2004). The Drosophila pericentrin-like protein is essential for cilia/flagella function, but appears to be dispensable for mitosis. *J. Cell Biol.* 165, 673-683. 10.1083/jcb.200402130 15184400 PMC2172389

[JCS194621C35] Mastronarde D. N. (2005). Automated electron microscope tomography using robust prediction of specimen movements. *J. Struct. Biol.* 152, 36-51. 10.1016/j.jsb.2005.07.007 16182563

[JCS194621C36] Nachury M. V., Seeley E. S. and Jin H. (2010). Trafficking to the ciliary membrane: how to get across the periciliary diffusion barrier? *Annu. Rev. Cell Dev. Biol.* 26, 59-87. 10.1146/annurev.cellbio.042308.113337 19575670 PMC2952038

[JCS194621C37] Nigg E. A. and Raff J. W. (2009). Centrioles, centrosomes, and cilia in health and disease. *Cell* 139, 663-678. 10.1016/j.cell.2009.10.036 19914163

[JCS194621C38] Phillis R. W., Bramlage A. T., Wotus C., Whittaker A., Gramates L. S., Seppala D., Farahanchi F., Caruccio P. and Murphey R. K. (1993). Isolation of mutations affecting neural circuitry required for grooming behavior in Drosophila melanogaster. *Genetics* 133, 581-592. 8454205 10.1093/genetics/133.3.581 PMC1205345

[JCS194621C39] Port F., Chen H.-M., Lee T. and Bullock S. L. (2014). Optimized CRISPR/Cas tools for efficient germline and somatic genome engineering in Drosophila. *Proc. Natl. Acad. Sci. USA* 111, E2967-E2976. 10.1073/pnas.1405500111 25002478 PMC4115528

[JCS194621C40] Reiter J. F., Blacque O. E. and Leroux M. R. (2012). The base of the cilium: roles for transition fibres and the transition zone in ciliary formation, maintenance and compartmentalization. *EMBO Rep.* 13, 608-618. 10.1038/embor.2012.73 22653444 PMC3388784

[JCS194621C41] Rogowski M., Scholz D. and Geimer S. (2013). Chapter Fourteen – Electron microscopy of flagella, primary cilia, and intraflagellar transport in flat-embedded cells. *Methods Enzymol.* 524, 243-263. 10.1016/B978-0-12-397945-2.00014-7 23498744

[JCS194621C42] Roque H., Wainman A., Richens J., Kozyrska K., Franz A. and Raff J. W. (2012). Drosophila Cep135/Bld10 maintains proper centriole structure but is dispensable for cartwheel formation. *J. Cell Sci.* 125, 5881-5886. 10.1242/jcs.113506 22976301

[JCS194621C43] Rubin G. M. and Spradling A. C. (1982). Genetic transformation of Drosophila with transposable element vectors. *Science* 218, 348-353. 10.1126/science.6289436 6289436

[JCS194621C44] Sang L., Miller J. J., Corbit K. C., Giles R. H., Brauer M. J., Otto E. A., Baye L. M., Wen X., Scales S. J., Kwong M. et al. (2012). Mapping the Nephronophtisis-Jouber-Meckel-Gruber protein network reveals ciliopathy disease genes and pathways. *Cell* 145, 513-528. 10.1016/j.cell.2011.04.019 PMC3383065 21565611

[JCS194621C45] Schindelin J., Arganda-Carreras I., Frise E., Kaynig V., Longair M., Pietzsch T., Preibisch S., Rueden C., Saalfeld S., Schmid B. et al. (2012). Fiji: an open-source platform for biological-image analysis. *Nat. Methods* 9, 676-682. 10.1038/nmeth.2019 22743772 PMC3855844

[JCS194621C46] Schouteden C., Serwas D., Palfy M. and Dammermann A. (2015). The ciliary transition zone functions in cell adhesion but is dispensable for axoneme assembly in C. elegans. *J. Cell Biol.* 210, 35-44. 10.1083/jcb.201501013 26124290 PMC4493997

[JCS194621C47] Seeds A. M., Ravbar P., Chung P., Hampel S., Midgley F. M., Mensh B. D. and Simpson J. H. (2014). A suppression hierarchy among competing motor programs drives sequential grooming in Drosophila. *Elife* 3, e02951 10.7554/eLife.02951 25139955 PMC4136539

[JCS194621C48] Slaats G. G., Isabella C. R., Kroes H. Y., Dempsey J. C., Gremmels H., Monroe G. R., Phelps I. G., Duran K. J., Adkins J., Kumar S. A. et al. (2015). MKS1 regulates ciliary INPP5E levels in Joubert syndrome. *J. Med. Genet.* 53, 62-72. 10.1136/jmedgenet-2015-103250 26490104 PMC5060087

[JCS194621C49] Stepanek L. and Pigino G. (2016). Microtubule doublets are double-track railways for intraflagellar transport trains. *Science* 352, 721-724. 10.1126/science.aaf4594 27151870

[JCS194621C50] Styczynska-Soczka K. and Jarman A. P. (2015). The Drosophila homologue of Rootletin is required for mechanosensory function and ciliary rootlet formation in chordotonal sensory neurons. *Cilia* 4, 9 10.1186/s13630-015-0018-9 26140210 PMC4489026

[JCS194621C51] Sung C.-H. and Leroux M. R. (2013). The roles of evolutionarily conserved functional modules in cilia-related trafficking. *Nat. Cell Biol.* 15, 1387-1397. 10.1038/ncb2888 24296415 PMC4016715

[JCS194621C52] Tammachote R., Hommerding C. J., Sinders R. M., Miller C. A., Czarnecki P. G., Leightner A. C., Salisbury J. L., Ward C. J., Torres V. E., Gattone V. H. et al. (2009). Ciliary and centrosomal defects associated with mutation and depletion of the Meckel syndrome genes MKS1 and MKS3. *Hum. Mol. Genet.* 18, 3311-3323. 10.1093/hmg/ddp272 19515853 PMC2733821

[JCS194621C53] Vannuccini E., Paccagnini E., Cantele F., Gentile M., Dini D., Fino F., Diener D., Mencarelli C. and Lupetti P. (2016). Two classes of short intraflagellar transport train with different 3D structures are present in Chlamydomonas flagella. *J. Cell Sci.* 129, 2064-2074. 10.1242/jcs.183244 27044756 PMC6518222

[JCS194621C54] Varmark H., Llamazares S., Rebollo E., Lange B., Reina J., Schwarz H. and Gonzalez C. (2007). Asterless is a centriolar protein required for centrosome function and embryo development in Drosophila. *Curr. Biol.* 17, 1735-1745. 10.1016/j.cub.2007.09.031 17935995

[JCS194621C55] Weatherbee S. D., Niswander L. A. and Anderson K. V. (2009). A mouse model for Meckel syndrome reveals Mks1 is required for ciliogenesis and Hedgehog signaling. *Hum. Mol. Genet.* 18, 4565-4575. 10.1093/hmg/ddp422 19776033 PMC2773271

[JCS194621C56] Wei Q., Zhang Y., Li Y., Zhang Q., Ling K. and Hu J. (2012). The BBSome controls IFT assembly and turnaround in cilia. *Nat. Cell Biol.* 14, 950-957. 10.1038/ncb2560 22922713 PMC3434251

[JCS194621C57] Williams C. L., Winkelbauer M. E., Schafer J. C., Michaud E. J. and Yoder B. K. (2008). Functional redundancy of the B9 proteins and nephrocystins in Caenorhabditis elegans ciliogenesis. *Mol. Biol. Cell.* 19, 2154-2168. 10.1091/mbc.E07-10-1070 18337471 PMC2366840

[JCS194621C58] Williams C. L., Li C., Kida K., Inglis P. N., Mohan S., Semenec L., Bialas N. J., Stupay R. M., Chen N., Blacque O. E. et al. (2011). MKS and NPHP modules cooperate to establish basal body/transition zone membrane associations and ciliary gate function during ciliogenesis. *J. Cell Biol.* 192, 1023-1041. 10.1083/jcb.201012116 21422230 PMC3063147

[JCS194621C59] Williams C. L., McIntyre J. C., Norris S. R., Jenkins P. M., Zhang L., Pei Q., Verhey K. and Martens J. R. (2014). Direct evidence for BBSome-associated intraflagellar transport reveals distinct properties of native mammalian cilia. *Nat. Commun.* 5, 5813 10.1038/ncomms6813 25504142 PMC4284812

[JCS194621C60] Winkelbauer M. E., Schafer J. C., Haycraft C. J., Swoboda P. and Yoder B. K. (2005). The C. elegans homologs of nephrocystin-1 and nephrocystin-4 are cilia transition zone proteins involved in chemosensory perception. *J. Cell Sci.* 118, 5575-5587. 10.1242/jcs.02665 16291722

[JCS194621C61] Yan Z., Zhang W., He Y., Gorczyca D., Xiang Y., Cheng L. E., Meltzer S., Jan L. Y. and Jan Y. N. (2013). Drosophila NOMPC is a mechanotransduction channel subunit for gentle-touch sensation. *Nature* 493, 221-225. 10.1038/nature11685 23222543 PMC3917554

[JCS194621C62] Yang T. T., Su J., Wang W.-J., Craige B., Witman G. B., Bryan Tsou M.-F. and Liao J.-C. (2015). Superresolution pattern recognition reveals the architectural map of the ciliary transition zone. *Sci. Rep.* 5, 14096 10.1038/srep14096 26365165 PMC4568515

[JCS194621C63] Yee L. E., Garcia-Gonzalo F. R., Bowie R. V., Li C., Kennedy J. K., Ashrafi K., Blacque O. E., Leroux M. R. and Reiter J. F. (2015). Conserved genetic interactions between ciliopathy complexes cooperatively support ciliogenesis and ciliary signaling. *PLOS Genet.* 11, e1005627 10.1371/journal.pgen.1005627 26540106 PMC4635004

[JCS194621C64] Young D. (1973). Fine structure of the sensory cilium of an insect auditory receptor. *J. Neurocytol.* 2, 47-58. 10.1007/BF01099207 4781454

[JCS194621C65] Zhang W., Yan Z., Jan L. Y. and Jan Y. N. (2013). Sound response mediated by the TRP channels NOMPC, NANCHUNG, and INACTIVE in chordotonal organs of Drosophila larvae. *Proc. Natl. Acad. Sci. USA* 110, 13612-13617. 10.1073/pnas.1312477110 23898199 PMC3746866

[JCS194621C66] Zhang Y., Seo S., Bhattarai S., Bugge K., Searby C. C., Zhang Q., Drack A. V., Stone E. M. and Sheffield V. C. (2014). BBS mutations modify phenotypic expression of CEP290-related ciliopathies. *Hum. Mol. Genet.* 23, 40-51. 10.1093/hmg/ddt394 23943788 PMC3857943

[JCS194621C67] Zhao C. and Malicki J. (2011). Nephrocystins and MKS proteins interact with IFT particle and facilitate transport of selected ciliary cargos. *EMBO J.* 30, 2532-2544. 10.1038/emboj.2011.165 21602787 PMC3155299

